# Increased P2X7 level does not affect anxiety or stress-coping behavior in male mice

**DOI:** 10.1007/s11302-026-10157-3

**Published:** 2026-05-09

**Authors:** Lidia Urbina-Treviño, Hao Tang, Iven-Alex von Mücke-Heim, Tobias Engel, Annette Nicke, Jan M. Deussing

**Affiliations:** 1 Molecular Neurogenetics, Max Planck Institute of Psychiatry, Kraepelinstraße 2-10, 80804 Munich, Germany; 2 Graduate School of Systemic Neurosciences, LMU Munich, Munich, Germany; 3 Graduate Center of Life Sciences, TUM School of Life Sciences, Munich, Germany; 4 Department of Physiology and Medical Physics, Royal College of Surgeons in Ireland, Dublin, D02 YN77 Ireland; 5 FutureNeuro, Research Ireland Centre for Chronic and Rare Neurological Diseases, RCSI University of Medicine and Health Sciences, Dublin, D02 YN77 Ireland; 6 Walther Straub Institute of Pharmacology and Toxicology, Faculty of Medicine, LMU Munich, Munich, Germany

**Keywords:** P2X7, P2RX7, Mood disorder, Depression, Overexpression, Mouse model

## Abstract

The *P2RX7* gene has been linked to various neuropsychiatric disorders. In particular, the SNP rs2230912, which results in a glutamine-to-arginine substitution at position 460, has repeatedly been associated with mood disorders. Although this SNP per se does not affect receptor function, it tags a gain-of-function haplotype that has been shown to significantly enhance receptor activity. BAC-transgenic P2X7-reporter mice have proven to be valuable tools for monitoring P2X7 expression. Here, we exploited their capacity to simultaneously overexpress the receptor, thereby serving as gain-of-function models to assess the behavioral consequences of elevated P2X7 levels. We used the two currently available transgenic P2X7 reporter lines (sEGFP and P2X7-EGFP), which differ in their expression pattern, degree of overexpression, and co-expression of the neighbouring *P2rx4* gene. Male sEGFP and P2X7-EGFP mice showed no alterations in general activity or in measures of anxiety-related or stress-coping behavior compared to their wild-type littermates. Only male P2X7-EGFP mice exhibited slightly delayed locomotor habituation to a novel environment. To what extent higher expression levels reflect enhanced receptor activity, as conveyed by the disease-associated gain-of-function haplotype, requires further investigation. Overall, these results indicate that P2X7 overexpression—whether at endogenous or ectopic sites, or in conjunction with P2X4—is not sufficient to substantially alter behavior of individually housed male mice under baseline conditions.

## Introduction

P2X7 is a ligand-gated ion channel that has been shown to play an important role in immune responses, inflammation, and cell death [1]. As a member of the P2X receptor family, it is activated by extracellular adenosine triphosphate (ATP). ATP binding opens a nonselective cation channel that allows K⁺ efflux and Na⁺/Ca^2^⁺ influx [2]. Compared to other family members, P2X7 is characterized by its unique large intracellular domain and a significantly lower affinity for ATP, suggesting that it is primarily activated at high or pathological concentrations of ATP when it primarily acts as a danger signal [3].

Candidate gene studies have identified single-nucleotide polymorphisms (SNPs) in the *P2RX7* gene as being associated with various psychiatric and neurologic disorders [4, 5]. In the *P2RX7* gene, numerous coding SNPs have been identified which confer complete or partial loss of function, gain of function, or have no immediate functional consequences. Particularly, the SNPs rs208294 (His155Tyr), rs7958311 (His270Arg), and rs1718119 (Ala348Thr) have been demonstrated to enhance ion channel function and pore formation [6, 7]. Interestingly, the SNP most frequently associated with mood disorders—rs2230912 (Gln460Arg)—does not affect receptor function when individually introduced into the wild-type P2X7 peptide sequence [6, 8]. However, co-expression of wild-type human P2X7 with the mutant P2X7-Arg460 variant has been demonstrated to significantly compromise receptor activity with respect to calcium influx, channel currents and intracellular signalling [8]. Based on this observation, humanized mice have been generated in which the murine *P2rx7* gene has been engineered to express wild-type human P2X7 or the P2X7-Arg460 variant [9]. Interestingly, particularly heterozygous mice co-expressing both variants showed reduced sleep quality and a higher vulnerability to chronic stress [10]. Notably, rs2230912 is part of the P2X7-4 haplotype, which comprises the gain-of-function alleles Tyr155, Arg270, and Thr348, and therefore serves as a tag SNP for a gain-of-function haplotype that collectively may contribute to the development of mood disorders [7, 11].

In the central nervous system (CNS), P2X7 is expressed in micro- and macroglia, whereas its expression in neurons has been a matter of controversy for some time. However, mouse genetic tools [9] and the availability of single-cell mRNA sequencing data clearly support its expression in different neuronal populations (compare e.g., http://mousebrain.org) [12]. Nevertheless, the broad and low expression renders its detection in brain tissue by immunohistochemistry rather challenging. To overcome this difficulty P2X7 reporter lines have been generated enabling the visualization of P2X7-expressing cells in the brain or the detection of P2X7 itself.

**Fig. 1:**
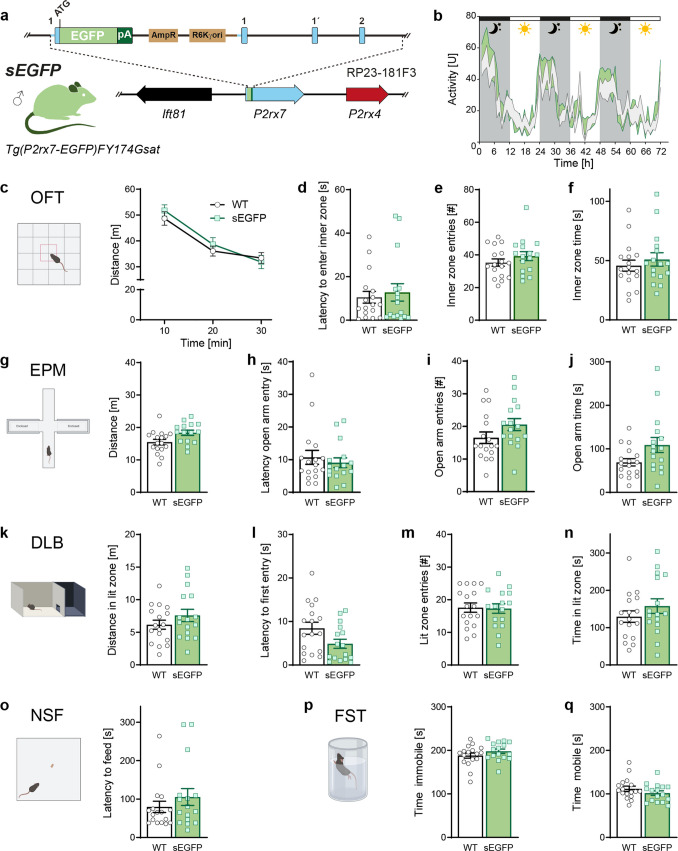
Behavioral characterization of sEGFP mice. **a** Schematic illustration of BAC transgenic construct present in sEGFP mice. AmpR: ampicillin resistance, R26Kγ ori: origin of replication. **b** Home cage activity monitored for 72 h. **c**–**f** Assessment of locomotion and anxiety-related behavior in the open field test (OFT), **g**–**j** elevated plus maze (EPM), **k**–**n** dark/light box (DLB) and **o** in the novelty suppressed feeding (NSF) paradigm. **p**, **q** Evaluation of stress-coping behavior in the forced swim test (FST). Data are presented as mean ± S.E.M. (WT *n* = 17, sEGFP *n* = 16). Illustrations of behavioral tests were created with BioRender.com/ywzsq7x

Using bacterial artificial chromosome (BAC) technology, two transgenic mouse models have been developed in the past to enable the visualization of P2X7 expression through enhanced green fluorescent protein (EGFP) controlled by regulatory sequences of the murine *P2rx7* gene [13]. A first line was generated as part of the Gene Expression Nervous System Atlas (GENSAT) initiative [14]. In this mouse line, a soluble form of EGFP is expressed under the control of the murine *P2rx7* promoter (Tg(P2rx7-EGFP)FY174Gsat/Mmuc; referred to as sEGFP). The sEGFP line has served as a reporter for monitoring P2X7 distribution in normal physiology and disease settings [15–17]. However, it has been demonstrated that the pattern of EGFP expression does not faithfully mirror native P2X7 localization [18]. In addition, the BAC recombineering process has generated a partial duplication of the *P2rx7* gene within the transgenic construct, which results in an unintended overexpression of P2X7. Moreover, the utilized BAC (RP23-181F3) comprises the neighbouring *P2rx4* gene, resulting in co-overexpression of P2X4 in this reporter line [18] (Fig. 1a).

**Fig. 2:**
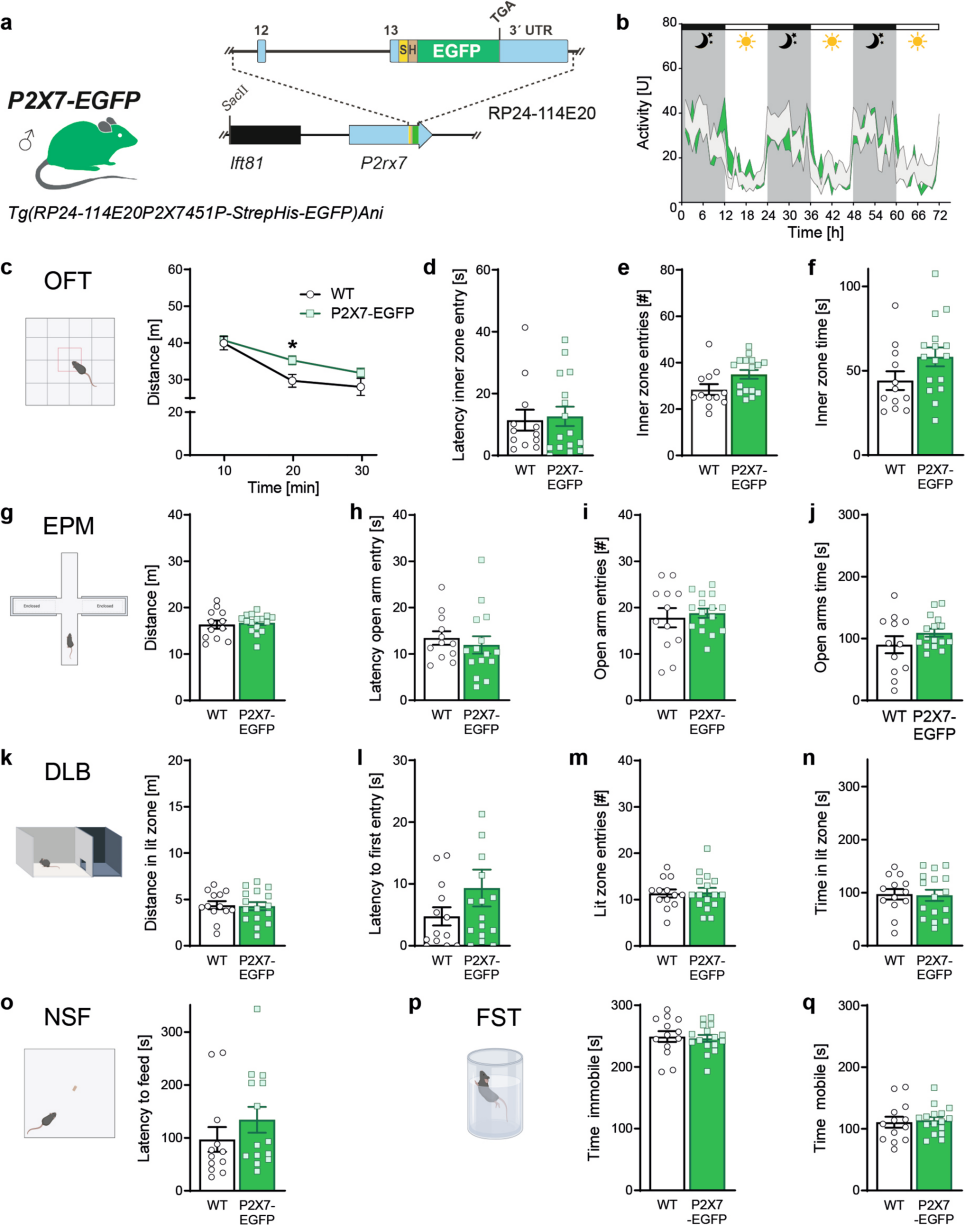
Behavioral characterization of P2X7-EGFP mice. **a **Schematic illustration of BAC transgenic construct present in P2X7-EGFP mice. S: Strep-tag, H: His-tag. **b** Home cage activity monitored for 72 h. **c**–**f **Assessment of locomotion and anxiety in the open field test (OFT), **g-j** elevated plus maze (EPM), **k**–**n** dark/light box (DLB), and **o** in the novelty suppressed feeding (NSF) test. **p**, **q** Evaluation of stress-coping behavior in the forced swim test (FST). * *p* = 0.0467, Bonferroni´s post hoc test. Data are presented as mean ± S.E.M. (in **b**, **k**–**n**, **p**, **q**: WT *n* = 13, P2X7-EGFP *n* = 16; in **c**–**j**: WT *n* = 12, P2X7-EGFP: *n* = 16; in **o**: WT *n* = 12, P2X7-EGFP *n* = 14). Illustrations of behavioral tests were created with BioRender.com/ywzsq7x

In the second BAC-based model (Tg(RP24-114E20P2X7-StrepHis-EGFP)Ani; referred to as P2X7-EGFP), EGFP is fused to the C-terminus of P2X7, allowing direct visualization of the tagged receptor (Fig. 2a). The expression profile in this model closely resembles endogenous P2X7 distribution, particularly within glial populations of the CNS [19]. The employed BAC clone (RP24-114E20) lacks the *P2rx4* locus and thus avoids co-expression of exogenous P2X4. The most thoroughly characterized reporter line carries 15 copies of the transgene, resulting in a substantial overexpression of P2X7 at endogenous sites [19].

In the present study, we attempted to model the mood disorder-associated gain-of-function haplotype by using mouse models of P2X7 overexpression. We utilized both BAC-transgenic reporter strains to investigate how elevated P2X7 expression, either at physiological sites or ectopically, as well as concomitant P2X4 overexpression, influences behavioral outcomes.

## Materials and methods

### Animals

Mice were group-housed under standard laboratory conditions and were maintained on a 12:12 h light–dark cycle (lights on from 07:00 to 19:00 h), with food and water provided ad libitum. The Tg(P2rx7-EGFP)FY174Gsat mouse line (sEGFP) was obtained from the Mutant Mouse Resource & Research Center. The Tg(RP24-114E20P2X7451P-StrepHis-EGFP)Ani mouse line (P2X7-EGFP, line 17) has been previously described (Kaczmarek-Hajek et al., 2018; Ramírez-Fernández et al., 2020). Experimental mice were kept on a C57BL/6N background and generated by breeding transgenic males to C57BL/6N females. To minimize stress caused by maintenance of social hierarchy and territorial behavior, male animals were single-housed prior to behavioral testing. Because single housing represents a significant stressor for female mice—and would add further variability due to fluctuating estrogen levels across the estrous cycle—we limited this study to male animals.

### Behavioral experiments

Behavioral testing was conducted between 8:30 a.m. and 12:30 p.m. during the light cycle. Male mice were single-housed 2 weeks before behavioral testing. Male littermates aged 12–16 weeks were employed in all tests.

#### Home cage activity

The home-cage activity was measured with Mouse-E-Motion infrared-detecting devices (Infra-e-motion, Germany). Mice were single-housed in fresh cages, and a plexiglass food tray was employed to hold the devices in place. The base bedding was kept, but extra materials that could conceal the animal were removed. The readout lasted 5–7 days during which time animals were not disturbed. Locomotor activity was detected in 4-min increments and averaged by the hour. The final analysis was applied to 3–4 full days, which started at least 24 h after the beginning of the test.

#### Open field test (OFT)

Exploratory behavior in a novel environment was investigated in the OFT. Mice were placed in a corner of a dimly lit (< 30 lx) open-field arena (50 cm × 50 cm × 40 cm) and allowed to freely explore the apparatus for 30 min. Anxiety-related parameters (latency to enter, number of entries and time spent in the inner zone) were assessed in the first 10 min of the OFT.

#### Dark/light box (DLB)

The DLB was conducted in a rectangular apparatus divided into a smaller dark zone (15 cm × 25 cm) and a larger illuminated zone (30 cm × 25 cm) connected by a small opening (5 cm × 7 cm). The lit zone was illuminated to 700 lx to create a highly aversive environment. Mice were initially placed in the dark zone and allowed to freely explore the apparatus for 10 min.

#### Elevated plus maze (EPM)

The EPM was performed in a plus-shaped elevated maze (30 cm) with two opposite open arms (5 × 30 cm), two closed arms (30 × 5 × 15 cm) and a central zone (5 × 5 cm). The illumination in the open arms was 30 lx and < 10 lx in the closed arms. Mice were placed in the central zone facing one of the closed arms and were allowed to freely explore the maze for 10 min.

#### Novelty suppressed feeding (NSF)

Mice were fasted overnight before the start of the experiment. Mice were placed in a novel environment (open-field arena, 50 × 50 × 50 cm) facing the corner with a food pellet presented in the middle of the arena, which was illuminated with more than 400 lx. Mice were allowed to explore the arena for 10 min, and the latency to feed was recorded.

#### Forced swim test (FST)

Active versus passive stress-coping behavior was assessed using the FST. The test was performed using a glass beaker (radius 11 cm, height 23.5 cm) filled with 1.5 l water (25 °C). Mice were subjected to a 6-min test, during which the time spent mobile and immobile was scored manually.

### Data analysis

Behavioral data were recorded and analyzed using ANY-Maze (Stoelting Co., Wood Dale, USA). Statistical analysis was carried out using GraphPad Prism v10.0 (GraphPad Software, La Jolla, CA, USA). Sample size was determined a priori assuming a standardized effect size of Cohen’s *d* = 1.2, corresponding to a difference of 1.2 pooled standard deviations between wild-type and transgenic mice, with a type I error rate of 0.05 and power of 80%. Normality was assessed using the Shapiro–Wilk test. Parametric data were analyzed by use of Student’s *t*-test or with a two-way analysis of variance (ANOVA) with repeated measures followed by a Bonferroni post hoc test. Data are presented as mean ± S.E.M. Statistical significance was defined as *p* < 0.05. The following data points were excluded in Fig. 2, for both the OFT (Fig. 2c–f) and EPM (Fig. 2g–j), one WT animal was excluded because it jumped out of the test apparatus. In the NSF test (Fig. 2o), one WT animal was not tested because of health issues on the day of testing, and two P2X7-EGFP animals were excluded from the analysis as they did not approach the food pellet. In Fig. 2h, one P2X7-EGFP data point was excluded after being identified as an outlier using Grubbs’ test.

## Results

To investigate the behavioral consequences of P2X7 overexpression, male sEGFP and P2X7-EGFP mice were subjected to a battery of tests assessing general activity, locomotion, anxiety-related and stress-coping behavior.

sEGFP mice did not show any alterations in general activity compared to control littermates when monitored over 3 nights and days (Fig. 1b). In the OFT, sEGFP mice were indistinguishable from their WT littermates with regard to the distance travelled and their ability to habituate to the novel environment of the test arena (Fig. 1c). Measures of anxiety-related behavior were unaffected in sEGFP mice, as indicated by the latency to enter, the number of entries and the time spent in the inner zone of the OF (Fig. 1d–f). In addition, we used the EPM as a standard paradigm to assess anxiety-related behavior. sEGFP and WT mice travelled a similar distance in the test apparatus and were indistinguishable with regard to the latency to enter, the number of entries and the time spent in the aversive open arms (Fig. 1g–j). The absence of an anxiety-related phenotype was further confirmed in the DLB. sEGFP and WT mice travelled a comparable distance in the lit compartment and showed no significant differences with regard to the latency to enter, the number of entries and the time spent in the lit compartment (Fig. 1k–n). We used the NSF test as a more locomotion-independent anxiety test but again did not observe any difference between sEGFP and WT animals when assessing the latency to feed (Fig. 1o). Finally, we used the FST to assess the coping strategy of sEGFP mice in a stressful situation. We did not detect any significant differences in the FST between sEGFP and WT mice when evaluating the time immobile and mobile, respectively (Fig. 1p, q).

Subsequently, we tested P2X7-EGFP mice overexpressing an EGFP-tagged variant of murine P2X7 in the same behavioral test battery. P2X7-EGFP mice and WT littermates showed comparable levels of general activity continuously monitored for 3 nights and days (Fig. 2b). In the 30-min OFT test, P2X7-EGFP mice showed a significantly increased distance travelled between 10 and 20 min (two-way ANOVA-RM; interaction, *F*_(2,52)_ = 3.377, *p *= 0.0418; genotype, *F*_(1,26)_ = 2.934, *p* = 0.0986, time, *F*_(2,52)_ = 67.73, *p* < 0.0001). However, measures of anxiety-related behavior such as the latency to enter, the number of entries and the time spent in the inner zone of the OFT were indistinguishable compared to WT mice (Fig. 2c–f). In the EPM, no difference in the distance travelled was detectable. Similarly, P2X7-EGFP mice were indistinguishable from their WT littermates with regard to the latency to enter, the number of entries and the time spent in the aversive open arm (Fig. 2g–j). Likewise, both genotypes did not differ in the latency to enter, the number of entries and the time spent in the lit compartment or the distance travelled in the lit compartment of the DLB (Fig. 2k–n). In the NSF test, we observed no difference between P2X7-EGFP and WT mice with regard to the latency to feed (Fig. 2o). Mice of both genotypes showed similar coping strategies in the FST, as indicated by comparable times spent immobile and mobile (Fig. 2, q).

## Discussion

Human genetic studies provide substantial evidence suggesting an association of SNPs in the *P2RX7* gene with various neuropsychiatric disorders. This is further supported by a brain proteome-wide association study which also identified P2X7 as causally linked to depression [20]. In particular, the gain-of-function haplotype P2X7-4, tagged by rs2230912, has repeatedly been associated with mood disorders [5, 7, 11]. The P2X7-4 haplotype-specific variants His155Tyr, His270Arg, and Ala348Thr have been demonstrated to individually enhance receptor activity and to act synergistically when introduced in combination [6, 7, 21]. In general, gain-of-function mutations can directly increase the activity of a protein, as demonstrated for P2X7 (e.g., with respect to ATP-induced currents and ethidium uptake) and many other proteins [7, 22–25]. Alternatively, gain-of-function mutations may affect regulatory sequences, which result in upregulation of gene expression [26, 27]. Both types of gain-of-function mutations may eventually have similar consequences on downstream signalling pathways and the manifestation of phenotypic alterations [28, 29]. Therefore, we hypothesized that the previously revealed increase in P2X7 protein levels in both reporter lines [18, 19] would, to some extent, mimic the higher P2X7 activity conferred by the P2X7-4 haplotype. It should be noted, however, that there is currently no functional evidence that P2X7 overexpression directly translates into increased receptor activity.

Comprehensive behavioral phenotyping of both reporter mouse lines did not reveal any significant differences with respect to general activity or endophenotypes of mood disorders when focusing on anxiety-related behavior and stress-coping strategies. The slightly enhanced locomotor activity shown by P2X7-EGFP compared to WT mice in the second 10-min bin of the OF most likely does not reflect a reduction in general locomotor activity, as there is no significant difference in the total distance travelled in the 30-min OF, but rather should be considered as a sign of a delayed habituation of P2X7-EGFP mice to the novel environment of the test. However, considering that this is the only parameter significantly deviating from WT behavior, further studies are necessary to confirm a causal relationship with P2X7 overexpression. Moreover, it has to be noted that with the current sample size the study was powered to detect large biologically relevant differences, and smaller effects may not have been detected. Previous studies investigating different P2X7 KO mouse lines revealed a rather heterogeneous and sometimes inconsistent picture with regard to the phenotypes in various behavioral assays observed across different laboratories [9, 30–36]. Although loss- and gain-of-function approaches address different biological questions, these earlier studies suggest that the overall effect of P2X7 on behavioral readouts under baseline conditions is rather moderate and accordingly sensitive to environmental factors such as differences in husbandry conditions, genetic background, breeding strategies, and exact testing procedures [37]. Accordingly, we tried to minimize environmental factors by focussing on male animals thereby reducing variability potentially caused by fluctuating estrogen levels across the estrous cycle. In addition, we single-housed males prior to behavioral testing which reduces consequences of social hierarchy and territorial behavior observed in group-housing conditions. However, the focus on male mice represents also a major limitation of the present study. Future studies will be necessary to extend these analyses to sex-specific effects of P2X7 overexpression and their contribution to behavioral phenotypes relevant to psychiatric disorders. Our results indicate that P2X7 overexpression, independently of whether it occurs at endogenous or ectopic sites, is not sufficient to affect the tested behavioral readouts. One possibility is that the constitutive overexpression throughout the lifespan activates compensatory mechanisms. The detection of profoundly increased P2X7 protein levels suggests that such mechanisms might rather involve an adaptive downregulation of downstream signalling mechanisms than of P2X7 expression itself [18, 19].

Nevertheless, we cannot rule out that the increased receptor activity conveyed by the gain-of-function haplotype might have functional consequences distinct from those related to mere overexpression. To more directly interrogate the P2X7-4 haplotype in vivo would require introducing the specific amino acid substitutions within the endogenous gene locus. Although the relevant amino acids are conserved between human and murine P2X7, one has to take into account the well-known species-specific pharmacological differences [38, 39]. Hence, a strategy of humanization as previously used to compare human wild-type P2X7 with its P2X7-Arg460 variant would be advantageous [9, 10].

Interestingly, sEGFP mice not only overexpress P2X7 (~ 4-fold) but also co-overexpress P2X4 (~ 8-fold) [18], a circumstance which allows for the parallel assessment of a P2X4 gain-of-function situation. Of note, and similar to the overexpression of P2X7, there is currently no experimental evidence demonstrating whether P2X4 overexpression is directly associated with increased receptor activity. Previously, the consequences of P2X4 receptor activation have been addressed using ivermectin as a positive allosteric modulator of the P2X4 receptor. Ivermectin has been demonstrated to strongly affect anxiety- and stress-coping-related behavior. However, it turned out that these effects are largely independent of P2X4 as respective KO mice showed a comparable behavioral response to ivermectin as WT mice [40]. Our results indicate that P2X4 overexpression in conjunction with P2X7 does not have any major effect on the tested behavioral readouts. This finding is in line with studies using P2X4 KO mice demonstrating that the lack of P2X4 does not affect anxiety-related behavior in the OFT, EPM, or DLB [41].

Taken together, we used P2X7 reporter mice that simultaneously overexpress the receptor to model the gain-of-function configuration of the mood disorder-associated P2X7-4 haplotype. We demonstrate that overexpression of P2X7 alone—whether at endogenous or ectopic sites—or in combination with P2X4 overexpression is not sufficient to significantly alter anxiety-related or stress-coping behaviors of male mice in the behavioral assays employed under the conditions tested. These findings underscore the need for future studies that more precisely recapitulate the genetic configuration of the P2X7-4 haplotype in order to directly determine its potential contribution to psychiatric disorders.

## Data Availability

All data is provided within the manuscript.
